# Changes in red blood cell membrane structure in type 2 diabetes: a scanning electron and atomic force microscopy study

**DOI:** 10.1186/1475-2840-12-25

**Published:** 2013-01-28

**Authors:** Antoinette V Buys, Mia-Jean Van Rooy, Prashilla Soma, Dirk Van Papendorp, Boguslaw Lipinski, Etheresia Pretorius

**Affiliations:** 1 Unit of Microscopy and Microanalysis, University of Pretoria, Pretoria, South Africa; 2 Department of Physiology, Faculty of Health Sciences, University of Pretoria, ARCADIA, Pretoria, 0007, South Africa; 3 Joslin Diabetes Center, Harvard Medical School, Boston, USA

**Keywords:** Red blood cells, Atomic force microscopy, Scanning electron microscopy, Type 2 diabetes

## Abstract

Red blood cells (RBCs) are highly deformable and possess a robust membrane that can withstand shear force. Previous research showed that in diabetic patients, there is a changed RBC ultrastructure, where these cells are elongated and twist around spontaneously formed fibrin fibers. These changes may impact erythrocyte function. Ultrastructural analysis of RBCs in inflammatory and degenerative diseases can no longer be ignored and should form a fundamental research tool in clinical studies. Consequently, we investigated the membrane roughness and ultrastructural changes in type 2 diabetes. Atomic force microscopy (AFM) was used to study membrane roughness and we correlate this with scanning electron microscopy (SEM) to compare results of both the techniques with the RBCs of healthy individuals. We show that the combined AFM and SEM analyses of RBCs give valuable information about the disease status of patients with diabetes. Effectiveness of treatment regimes on the integrity, cell shape and roughness of RBCs may be tracked, as this cell’s health status is crucial to the overall wellness of the diabetic patient.

## Introduction

The erythrocyte is a unique anuclear cell, with a cytoplasm consisting of 95% hemoglobin, the protein responsible for oxygen transfer from the lungs to the rest of the body. For the erythrocyte to transport haemoglobin and subsequently oxygen to all cells, it must travel through the circulatory system, including the microcirculation, where it encounters small capillary spaces and high shear stresses. In order for the cell to be able to survive this, a highly deformable yet robust membrane is required. The erythrocyte membrane consists of an overlaying asymmetric phospholipid bilayer membrane, supported by an underlying spectrin-actin cytoskeletal complex which is interconnected by junctional complexes resulting in a simple hexagonal geometric matrix. The plasma membrane is anchored to the spectrin network mainly by proteins ankyrin and the transmembrane protein band 3 and 4.1 [1,2].

It is generally believed that the plasma membrane together with its cytoskeletal support is responsible for the maintenance of the shape and stability of the cell and also for allowing extensive deformations when needed [3]. Furthermore, the roughness of the cell membrane is a very interesting indicator of cell's health state [4]. Modifications of the lipid composition and the asymmetry of the bilayer have been shown to affect the overall shape of the erythrocyte and also the cell’s deformability. Alterations of the cytoskeletal proteins that connect the bilayer and the spectrin network, impact on particularly the erythrocyte membrane integrity, when encountering shear stresses [5]. Changes in the shape, mechanical characteristics or the integrity of the erythrocyte has severe implications on the functionality of the cell, as can be seen in several dysfunctional states of the erythrocyte, whether it is environmentally induced, due to hereditary defects or diseased states [3]. More specifically, researchers have suggested that the cell-membrane skeleton integrity measured as surface roughness is well correlated to the functional status of the cell, with a decrease of the membrane roughness seen in cells from diseased individuals [2,3]. Artificially induced injury to erythrocytes, shows a relationship between cytoskeleton integrity and membrane roughness. Other types of blood cells also show changes in membrane roughness during disease; for example, cell membranes of T-lymphocytes in thyroid associated ophthalmopathy [6].

Previous research by our team showed that in diabetic (Type 2) patients, there is a changed RBC ultrastructure, suggested to be caused by iron overload and subsequent non-enzymatic fibrinogen polymerization [7]. These changes are specifically due to inflammation in this condition, which is associated with thrombotic events [8,9]. Furthermore, diabetes is associated with disturbed erythrocyte membrane architecture and functions of erythrocytes at molecular scale may be compromised [10]. Previously, we have shown that in these patients, the RBCs are elongated and twist around spontaneously formed fibrin fibers when a RBC smear is studied. These fibrin fibers may become thickened matted fibrin masses and may be the cause of the thrombotic events [11] and we also noted that the RBCs intertwine with the fibrin and they have a changed membrane ultrastructure.

Due to this observation, the current study investigates the membrane roughness and changes in diabetes using atomic force microscopy (AFM). We correlate this with scanning electron microscopy (SEM) results and compare results from both the techniques with the RBCs of healthy individuals.

## Materials and methods

### Patients

Blood was drawn from 10 healthy individuals who were non-smoking and who did not take any medication. Red blood cell smears were compared to our micrograph bank consisting of hundreds of RBC micrographs and found to be representative of a typical RBC. No spontaneous fibrin fiber networks were found between these RBCs, suggesting that no inflammation is present. The diabetic group for the current study consisted of 10 diabetic patients recruited from the diabetic clinic. Their RBC smears were compared with out diabetic RBC database consisting of over 60 patients and found to be comparable. The group comprised of both males and females, with a longstanding history of diabetes, but clinically stable. There was also a high incidence of both macro- and micro vascular complications present among them. Treatment varied but most of the patients were on a combination of both insulin and oral hypoglycaemic agents. Ethical clearance was obtained for ultrastructural analysis form the University of Pretoria Human Ethics Committee and all participants completed informed consent forms.

### Sample preparation and viewing–SEM

Blood was collected in citrate tubes and a smear was made on glass cover slips. The cover slips were incubated for 5 min at 37°C followed by placement in a petri dish with Dulbecco's Phosphate buffered saline (DPBS) buffer. The samples where then washed for 20 min on a shaker to separate the RBCs from plasma proteins.

Washed samples were then fixed in 2.5% gluteraldehyde DPBS with a pH of 7.4 for 30 min. This was followed by rinsing thrice in buffer for 3 min before being fixed for 15 min with 1% osmium tetraoxide (OsO_4._) The samples were rinsed thrice with DPBS for 3 min and were dehydrated serially in 30%, 50%, 70%, 90% and 3 times with 100% ethanol. The SEM procedures were completed by drying of the material in hexamethyldisilazane (HMDS), followed by mounting and coating with carbon and examining using a ZEISS ULTRA Plus FEG-SEM.

### Sample preparation - AFM

1 ml of blood was drawn in citrate tubes and centrifuged at 1000 rpm for two minutes. The supernatant was discarded (plasma, platelets and white blood cells) and the remaining pellet (erythrocytes) was suspended in 2.5% glutaraldehyde for 30 min, rinsed with DPBS and post-fixed with OsO_4_ to ensure the preservation of membrane phospholipids. The samples were dehydrated with a series of ethanol and dried on a glass coverslip using HMDS.

#### AFM imaging and measurement

An AFM (Dimension Icon, Bruker, USA) was used in tapping mode to obtain topographic images. Aluminum coated nitride tips (TESPA, Bruker, USA) with a spring constant of 20 – 80 N/m, a resonant frequency between 382 – 405 kHz and a nominal tip radius of 8 nm was employed in all AFM measurements. Eight cells of each sample was scanned at the following fields; 10 μm by 10 μm (this is similar to a ± 25000–30000 times magnification) and 1000 nm by 1000 nm. NanoScope Analysis (Bruker, USA) was used to filter out any noise, subtract the plane of average inclination and perform all measurements. The following parameters were measured:

1. The macroparameters of the form and the size of cells were measured from the 10 μm by 10 μm scan; these measurements included height of the erythrocytes, the concave depth of the hollow and the diameter of the erythrocytes.

2. Membrane roughness was measured on the 1000 nm by 1000 nm scans after Fourier Transform (FT) was performed, to isolate three specific spatial domains each reflecting a typical erythrocyte membrane feature as shown in the literature [1,12]. The statistical significance of the difference between measurements was determined using one-way analysis of variance. A 2-tailed P value of less than 0.05 was considered significant.

## Results

**Figure 1:**
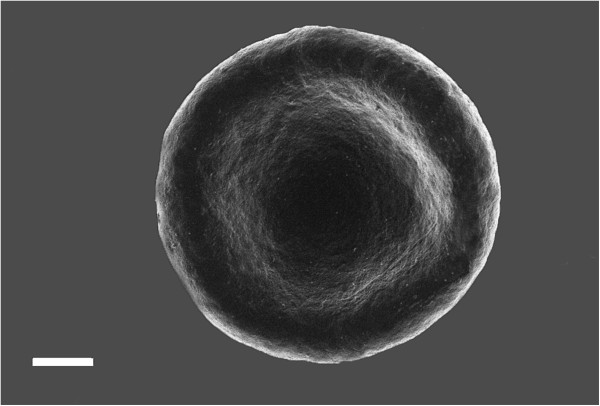
**SEM micrograph of an RBC from a healthy individual showing the typical morphology.** Scale = 1 μm.

**Figure 2:**
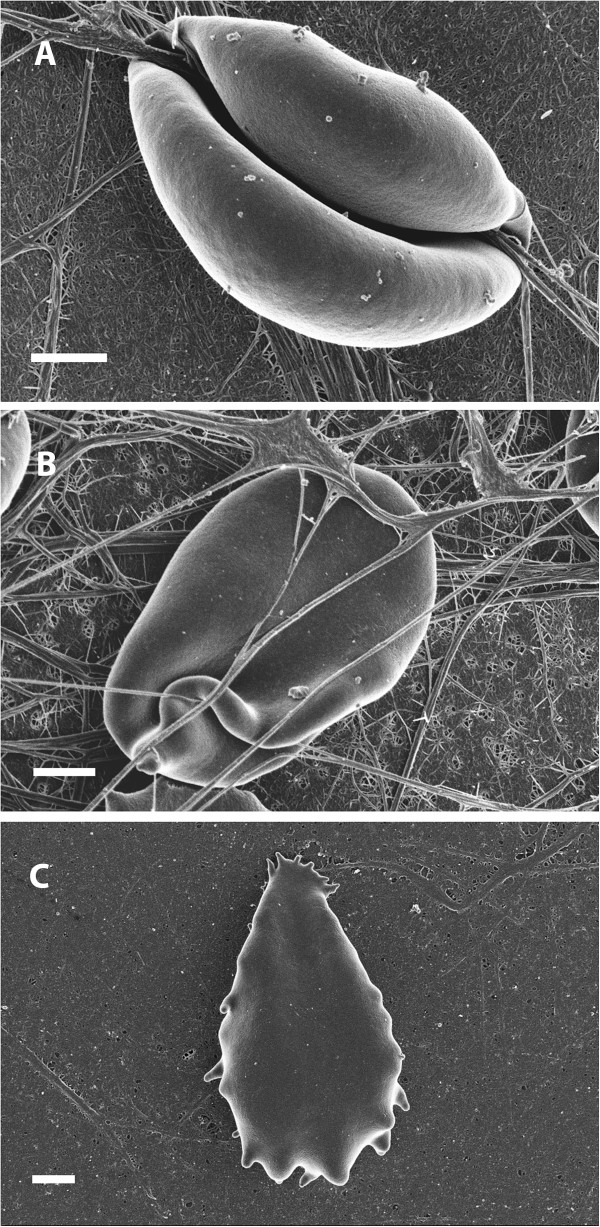
**SEM micrograph of an RBC of a diabetic individual. A**) RBC with very smooth membrane twisted around spontaneously formed fibrin fibers; **B**) RBC showing lengthened ultrastructure. **C**) RBC showing smooth membrane. Scale = 1 μm.

Figure 1 shows an RBC from a healthy individual and Figure 2A-C show RBCs from a diabetic patient. These results are comparable to previously published results [7]. In the healthy group, RBCs show the typical concave shape. However, in diabetes the RBCs are elongated and their membranes form extended projections and twist spontaneously around fibrin fibers (Figure 2A and B). Figure 2C shows an RBC with elongated shape and also a smooth membrane. Due to this phenomenon, we performed AFM analysis to determine the shape and membrane roughness with the AFM. Two smears of each patient was made and the 3 of the co-authors studied the samples to confirm that there were no differences between the two smears of each individual and that the micrographs taken from each individual compares to the RBCs of the whole group. The final micrographs chosen for this manuscript was confirmed to be representative of the whole sample. There seems to be no correlation between the deformation variability, amount of vascular complications and type of medication.

AFM measurement of the macroparameters indicated that erythrocytes from patients suffering from diabetes are smaller, with a reduced concave depth (Table 1). Measurement of the surface roughness of three orders of spatial domains contributing to the topography of the erythrocyte membrane is shown in Table 2. A decrease of roughness by about half, in erythrocytes of diabetic patients was noted.

**Table 1:** Macroparameter measurement results from healthy and diabetic erythrocytes

	**Control**	**Diabetic**	**p-value**
**Diameter**	7.22 μm ± 0.16 μm	6.80 μm ± 0.08 μm	0.028584*
**Height**	2.68 μm ± 0.04 μm	1.48 μm ± 0.11 μm	2.44057 E-07*
**Concave Depth**	358.2 nm ± 117.6 nm	153.9 nm ± 58.6 nm	0.00296363*

*All p-values indicated significant difference between two groups.

**Table 2:** Roughness measurement results from healthy and diabetic erythrocytes

	**Control**	**Diabetic**	**p-value**
**Roughness 1**^**st **^**order**	3.099 nm ± 0.499 nm	1.70 nm ± 0.13 nm	0.000141*
**Roughness 2**^**nd **^**order**	4.12 nm ± 0.51 nm	1.66 nm ± 0.24 nm	6.42 E-07*
**Roughness 3**^**rd **^**order**	1.72 nm ± 0.199 nm	0.82 nm ± 0.11 nm	0.000304*

*All p-values indicated significant difference between two groups.

**Figure 3:**
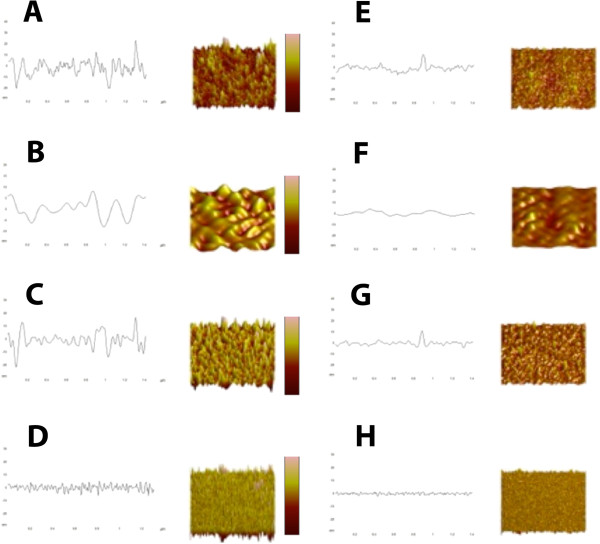
A) Control original profile and surface, z scale = 60nm, x and y scale = 1μm, B) Control first order profile and surface, z scale = 40nm, x and y scale = 1μm, C) Control second order profile and surface, z scale = 40nm, x and y scale = 1μm, D) Control third order profile and surface, z scale = 18nm, x and y scale = 1μm, E) Diabetes original profile and surface, z scale = 60nm, x and y scale = 1μm, F) Diabetes first order profile and surface, z scale = 40nm, x and y scale = 1μm, G) Diabetes second order profile and surface, z scale = 40nm, x and y scale = 1μm, H) Diabetes third order profile and surface, z scale = 18nm, x and y scale = 1μm.

Figure 3A-D shows the original topographical structure and line profile from a healthy individual from the sample, and also the topographical structure and line profiles of three separate spatial domains (isolated from the original image by means of FT), each illustrating specific membrane features. Figure 3E-H shows the same in the sample obtained from diabetic patients.

## Discussion

In the current analysis, a difference in diameter, height and concave depth between the healthy and diabetic individuals were seen (Table 1). This correlates with the SEM visual analysis, where the RBCs from diabetic patients differ in shape and size when compared to RBCs from healthy individuals (Figures 1 and 2).

It has been shown that the topographical nanostructure of the erythrocyte membrane and the roughness of these structures can be classified as independent morphological parameters of the membrane, describing both the primary and the altered structure and functional status of the membrane [1-3,13]. Furthermore, Space Fourier Transform allows for the isolation of different spectral periods that directly correlate to the different constituents of the erythrocyte membrane [1,12]. AFM is an ideal method to analyze the structure of the erythrocyte membrane, its main advantage being that qualitative images are accompanied by the corresponding quantitative height data, which allows for further measurements and analysis, for example Fourier Transform. The selection of the size of the spectral domains is guided by the structural properties of the erythrocyte membrane [1,12]. The first order surface of the erythrocyte membrane represents the accepted undulate nature of the erythrocyte membrane relating to the macroparameters of the cell, the second order surface correlate with the underlying spectrin-actin cytoskeletal complex and the third order surface represent the upper most protein, protein cluster and other membrane macromolecular topography (Figure 3).

Measurement of surface roughness (Table 2) indicated alterations in the first order surface of the cell, relating to the cells macro parameters, as is also seen in the macro parameter measurements, the roughness of the second order surface is also decreased in diabetic patients, indicating alterations in the cytoskeletal matrix and the connections between band 3 and 4 proteins and the matrix. A decrease in roughness measurements in the third order surface indicates superficial protein structure rearrangement. This correlates well with the SEM visual analysis, where the diabetes RBCs visibly appear smoother to that of the healthy RBCs (Figure 1 and 2).

It has been found that the cytoskeletal proteins of RBCs from diabetic patients are heavily glycosylated and that spectrin is oxidatively damaged [14]; also several lipids (free cholesterol, sphingomeyelin and phosphatidylcholine) on the outer surface of the phospholipid bilayer is significantly decreased [15,16]. This directly correlates with the ultrastructural roughness results seen by the AFM of the second and third order respectively.

We conclude by suggesting that the combined AFM and SEM analyses of RBCs might give valuable information about the disease status of patients with diabetes. The ultrastructural changes discussed here are not visible using a traditional light microscope. We believe that ultrastructural analysis of RBCs in inflammatory diseases can no longer be ignored and should form a fundamental research tool in clinical studies. Efficacy of treatment regimes on the integrity, cell shape and roughness and health status of RBCs may be tracked, as this cell’s health status is crucial to the overall wellness of the diabetic patient.

### Ethical approval disclosure

Ethical approval was granted at the University of Pretoria (HUMAN ETHICS COMMITTEE: FACULTY OF HEALTH SCIENCES) under the name of E Pretorius (corresponding author). All human blood samples obtained were analyzed at the University of Pretoria and all participants filled in informed consent forms.

## Competing interests

There are no competing interests for any of the authors.

## Authors’ contributions

AVB did AFM analysis; MVR prepared SEM and AFM samples PS and DVP are the medical practitioners that identified the patients and confirmed their diagnosis, BL and EP are the principal investigators and EP did the SEM analysis and prepared the figures. All authors were equally involved in the manuscript preparation. All authors have read and approved the final manuscript.
